# BRD4 inhibitors broadly promote erastin-induced ferroptosis in different cell lines by targeting ROS and FSP1

**DOI:** 10.1007/s12672-024-00928-y

**Published:** 2024-04-03

**Authors:** Chenyang Fan, Xiaohong Guo, Jie Zhang, Wen Zheng, Chonglin Shi, Yongwei Qin, Haoliang Shen, Yang Lu, Yihui Fan, Yanli Li, Liuting Chen, Renfang Mao

**Affiliations:** 1 Department of Pathogenic Biology, School of Medicine, Nantong University, Nantong, China; 2 The Intensive Care Unit, Affiliated Hospital of Nantong University, Jiangsu, China; 3 Laboratory of Medical Science, School of Medicine, Nantong University, Nantong, China; 4 Department of Pathophysiology, School of Medicine, Nantong University, Nantong, China

**Keywords:** Ferroptosis, BRD4, ROS, FSP1, JQ-1, I-BET-762

## Abstract

Ferroptosis, an iron-dependent form of programmed cell death, is a promising strategy for cancer treatment. Bromodomain-containing protein 4 (BRD4) is an epigenetic reader and a promising target for cancer therapeutics. However, the role of BRD4 in ferroptosis is controversial and the value of the interaction between BRD4 inhibitors and ferroptosis inducers remains to be explored. Here, we found that BRD4 inhibition greatly enhanced erastin-induced ferroptosis in different types of cells, including HEK293T, HeLa, HepG2, RKO, and PC3 cell lines. Knocking down *BRD4* in HEK293T and HeLa cells also promoted erastin-induced cell death. BRD4 inhibition by JQ-1 and I-BET-762 or *BRD4* knockdown resulted in substantial accumulation of reactive oxygen species (ROS) in both HEK293T and HeLa cells. The effect of BRD4 inhibition on ferroptosis-associated genes varied in different cells. After using BRD4 inhibitors, the expression of FTH1, Nrf2, and GPX4 increased in HEK293T cells, while the levels of VDAC2, VDAC3, and FSP1 decreased. In HeLa cells, the expression of FTH1, VDAC2, VDAC3, Nrf2, GPX4, and FSP1 was reduced upon treatment with JQ-1 and I-BET-762. Consistently, the level of FSP1 was greatly reduced in HEK293T and HeLa cells with stable *BRD4* knockdown compared to control cells. Furthermore, ChIP-sequencing data showed that BRD4 bound to the promoter of *FSP1*, but the BRD4 binding was greatly reduced upon JQ-1 treatment. Our results suggest that ROS accumulation and *FSP1* downregulation are common mechanisms underlying increased ferroptosis with BRD4 inhibitors. Thus, BRD4 inhibitors might be more effective in combination with ferroptosis inducers, especially in FSP1-dependent cancer cells.

**Supplementary Information:**

The online version contains supplementary material available at 10.1007/s12672-024-00928-y.

## Introduction

Ferroptosis is an iron-dependent form of programmed cell death, and differs from apoptosis, necrosis, and autophagy [1]. Research has found that ferroptosis is an important factor in the development of many diseases. Ferroptosis significantly induces cancer cell death, and therefore has been recognized as an important tumor-inhibiting mechanism [1]. Furthermore, ferroptosis is considered an effective treatment to avoid drug resistance of cancer cells [2]. Ischemia–reperfusion injury and degenerative lesions of cerebral nerves, including Alzheimer’s disease and Parkinson’s disease, are also considered to be associated with ferroptosis [2]. Thus, manipulation of ferroptosis might be a novel means to treat a variety of diseases.

Ferroptosis is a cell death program induced by lipid peroxidation of highly expressed unsaturated fatty acids on cell membranes catalyzed by ferric or ester oxygenase [3]. Ferroptosis is regulated by multiple factors. An increase in Fe^2+^ and reactive oxygen species (ROS) is an important factor in ferroptosis [3]. Erastin is a classical ferroptosis inducer, which promotes ROS accumulation to cause ferroptosis through voltage-dependent anion channel 2 (VDAC2) and VDAC3 [4]. Nuclear factor E2-related factor 2 (Nrf2) is a common intracellular antioxidant pathway [2]. Furthermore, glutathione peroxidase (GPX4) and ferroptosis suppressor protein 1 (FSP1) are the two main independent signaling pathways that inhibit ferroptosis by inhibiting ROS accumulation [2].

Bromodomain-containing protein 4 (BRD4), a member of the bromo and extra-terminal (BET) family, affects transcription and epigenetic inheritance [5]. BRD4 was found to be highly expressed in cancer tissues and is believed to be associated with poor prognosis in cancer patients [6]. In addition, BRD4 regulates the expression of multiple genes or pathways that are related to ferroptosis. For example, BRD4 alleviated renal ischemia–reperfusion injury by reducing ROS production [7]. BRD4 inhibited the expression of Nrf2 in some cells [8, 9], but increased the expression of Nrf2 in other cells [10]. These studies indicated a potential link between BRD4 and ferroptosis. Indeed, one study suggested that BRD4 may affect the development of cancer by inhibiting ferroptosis. JQ-1, an inhibitor of BRD4, showed an anticancer role by promoting ferroptosis in MDA-MB-231 and Hs578T cells (breast cancer cell lines), and in A549 cells (lung cancer cell line) [6]. However, a recent study revealed that BRD4 inhibitors block erastin-induced ferroptosis by weakening the oxidative catabolism in mitochondria [11]. Therefore, previous studies provided controversial conclusions and unclear downstream mechanisms. Here, we evaluated the effector of BRD4 inhibition in a panel of five cell lines. Our results consistently showed that BRD4 inhibition promoted ferroptosis. In different cell types, the downstream effects upon BRD4 inhibition slightly differed, but increased ROS generation and decreased VDAC2/FSP1 expression were common to cell types.

## Results

### BRD4 inhibitors and genetic silencing promoted ferroptosis induced by erastin in a panel of five cell lines, HEK293T, HeLa, HepG2, RKO, and PC3

**Fig. 1:**
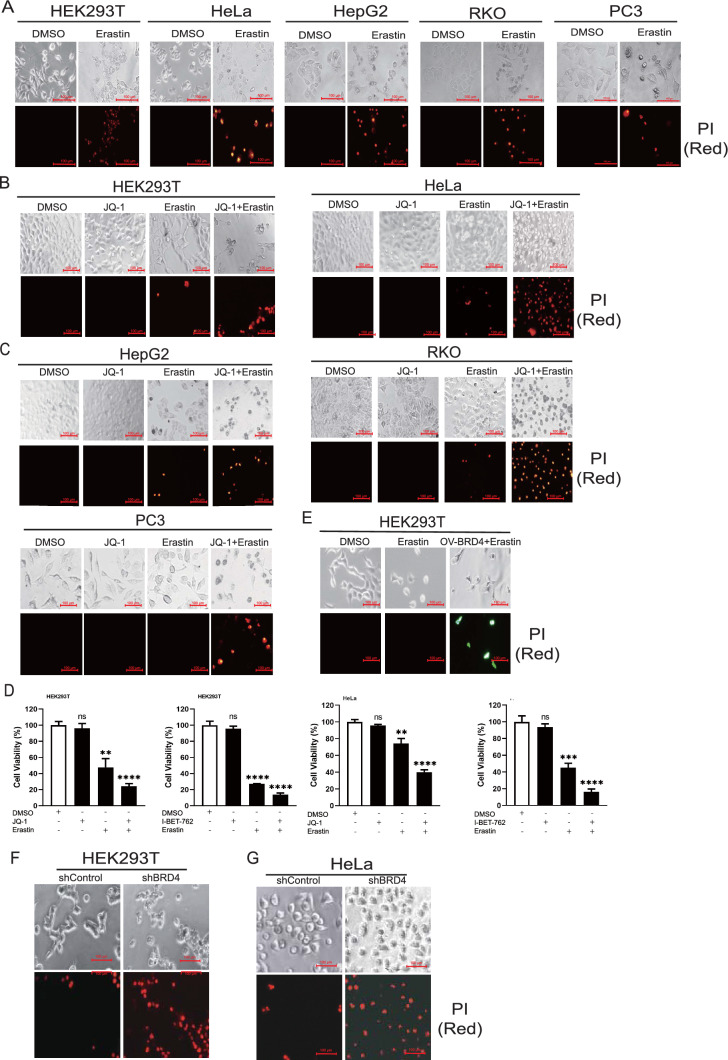
BRD4 alleviates ferroptosis induced by Erastin in a panel of five cell lines including HEK293T, HeLa, HepG2, RKO and PC3 cells. **A** Representative pictures and propidium iodide staining of HEK293T, HeLa, HepG2, RKO and PC3 cells after Erastin (20 μM) treatment for 24 h. Representative pictures and propidium iodide staining of (**B**) HEK293T cells, HeLa cells, (**C**) HepG2 cells, RKO cells and PC3 cells after being treated with DMSO, JQ-1 (1 μM), I-BET-762 (2 μM), Erastin (20 μM) or Erastin plus JQ-1, I-BET-762 respectively for 48 h. **D** CCK-8 was used to detect the cell viability of HEK293T cells and HeLa cells. **E** Representative pictures of HEK293T cells transfected with vector or plasmids encoding BRD4 after being treated with DMSO or Erastin (20 μM) respectively for 48 h. **F** Representative pictures of control and stable BRD4 knockdown cells in 293 T cells being treated with Erastin (20 μM) for 48 h. **G** Representative pictures of control and stable BRD4 knockdown cells in HeLa cells being treated with Erastin (20 μM) for 48 h. *p < 0 .05; **p < 0 .01; ***p < 0.001; ****p < 0.0001. Values are mean ± SD, n = 5

First, we investigated the effect of BRD4 inhibitors on ferroptosis. Erastin is a ferroptosis inducer, and it induces ferroptosis in HEK293T and HeLa cells as evidenced by the cellular morphology and propidium iodide (PI) staining (Fig. 1A). JQ-1 and I-BET-762 are inhibitors of BRD4 [6]. Upon BRD4 inhibition by JQ-1 and I-BET-762, erastin-induced ferroptosis was greatly enhanced in HEK293T cells (Fig. 1B and Additional file 1: Figure S1A). We obtained similar results by using HeLa cells (Fig. 1B and Additional file 1: Figure S1A). To validate these results, we performed similar experiments on additional cell lines, HepG2 (hepatocellular carcinoma), RKO (colon carcinoma), and PC3 (prostatic adenocarcinoma). The results in these three cell lines were similar to those in 293 T and HeLa cells (Fig. 1C and Additional file 1: Figure S1B, S1C, and S1D). Erastin-induced cell death was greatly enhanced by JQ-1 or I-BET-762 treatment. To further confirm these results, we quantified the cell viability. As shown, erastin treatment reduced cell viability to 50%, which was significantly further reduced to 20% when co-treated with JQ-1 (Fig. 1D). Similar results were observed in these cell lines when another inhibitor, I-BET-762, was used, as well as in HeLa cells (Fig. 1D). To examine whether BRD4 played a critical role, we overexpressed BRD4-GFP in HEK293T cells. Compared to GFP-negative cells, GFP-positive cells were more resistant to erastin (Fig. 1E). These results demonstrated that BRD4 inhibition promoted ferroptosis.

To exclude off-target effects of JQ-1 and I-BET-762, we generated stable *BRD4* knockdown in 293 T and HeLa cells. Both RT-PCR and western blot confirmed the knockdown efficiency (Figure S2A and S2B). Then, we treated the control and stable *BRD4* knockdown cells with erastin. As shown in Fig. 1F and 1G, erastin slightly induced cell death in control cells. However, in *BRD4* knockdown cells, the erastin-induced cell death was greatly increased. Together, our results indicated that inhibition of BRD4 greatly promoted erastin-induced cell death.

### Effect of BRD4 inhibitors on ferritin heavy chain 1 (FTH1) and ferritin light chain (FTL) in HEK293T and HeLa cells

**Fig. 2:**
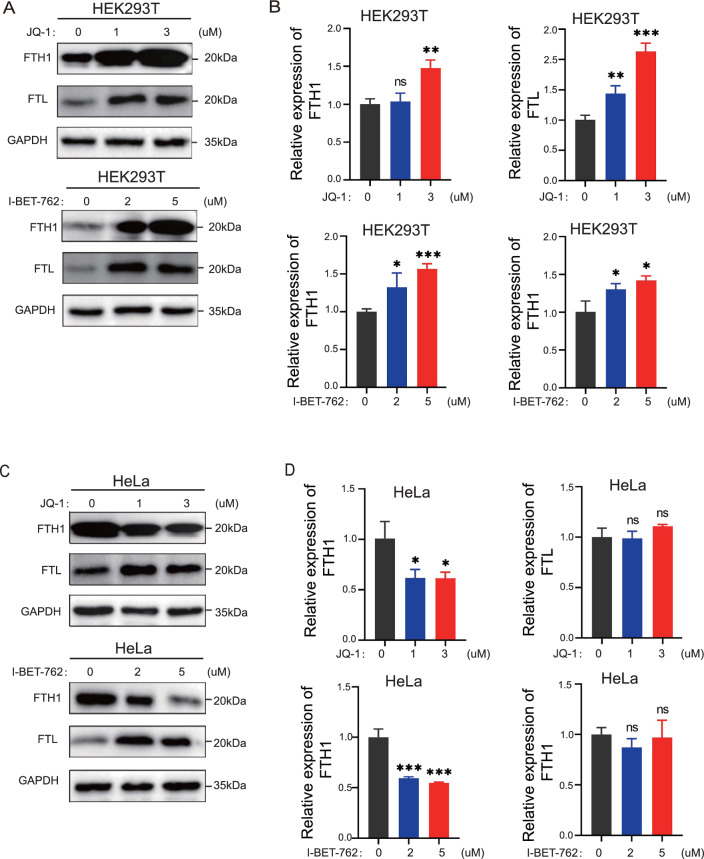
Effect of BRD4 on FTH1 and FTL in HEK293T cells and HeLa cells. HEK293T cells and HeLa cells were treated with JQ-1 and I-BET-762 for 24 h. **A** The protein levels of FTH1 and FTL in HEK293T cells were detected by Western blot. **B** qRT-PCR analysis of the mRNA expression levels of FTH1 and FTL in HEK293T cells. **C** The protein levels of FTH1 and FTL in HeLa cells were detected by Western blot. **D** qRT-PCR analysis of the mRNA expression levels of FTH1 and FTL in HeLa cells. *p < 0 .05; **p < 0 .01; ***p < 0.001

The accumulation of excess free Fe^2+^ in cells is an important factor in ferroptosis. FTH1 and FTL promote the storage of Fe^2+^ in cells and maintain the homeostasis of iron [12, 13]. To understand the molecular mechanism of BRD4 inhibition, we examined key pathways associated with ferroptosis. We found that BRD4 inhibition upregulated the protein levels of FTH1 and FTL in HEK293T cells (Fig. 2A). Meanwhile, BRD4 inhibitors also activated the mRNA expression of *FTH1* and *FTL* to varying degrees (Fig. 2B). In HeLa cells, both the protein and mRNA of FTH1 were downregulated after treatment with BRD4 inhibitors (Fig. 2C and 2D). However, FTL levels did not change significantly in HeLa cells. These studies suggest that BRD4 inhibitors downregulated FTH1 expression in certain cells. However, the reduced FTH1 expression upon BRD4 inhibition did not explain the common promoting effect on ferroptosis after BRD4 inhibition.

### BRD4 inhibitors or genetic silencing increased ROS and malondialdehyde levels of HEK293T and HeLa cells

**Fig. 3:**
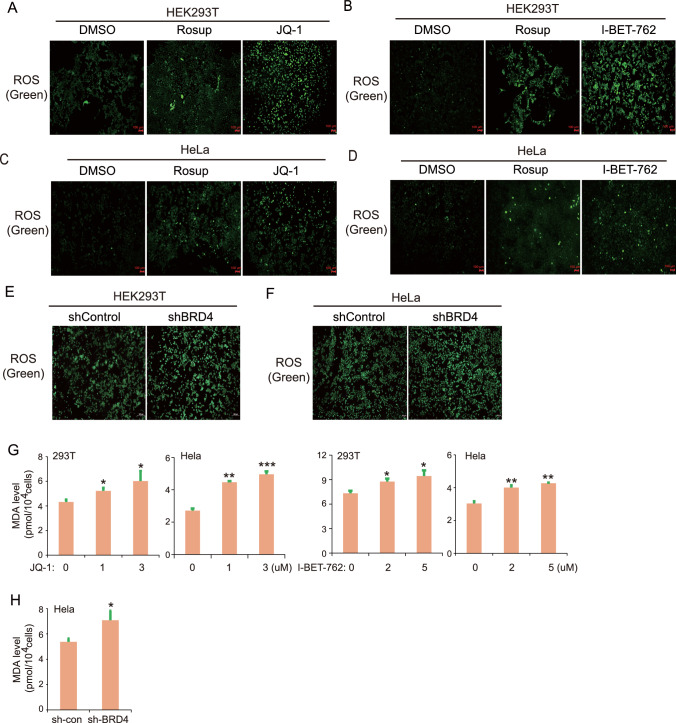
Effect of BRD4 on ROS levels in HEK293T cells and HeLa cells. HEK293T cells and HeLa cells were treated with JQ-1 (3 μM) and I-BET-762 (5 μM) respectively for 24 h. Rosup (50 μg/mL) was used as positive control to treat cells for 1 h. Reactive oxygen species assay kit was used to assess the ROS level in cells. Representative pictures of ROS level in HEK293T cells after being treated with (**A**) JQ and **B** I-BET-762. Representative pictures of ROS level in HeLa cells after being treated with (**C**) JQ and **D** I-BET-762. **E** Representative pictures of ROS level in control and stable BRD4 knockdown cells in 293 T cells. **F** Representative pictures of ROS level in control and stable BRD4 knockdown cells in HeLa cells. **G** 293T and Hela cells were treated with JQ-1 or I-BET-762. The level of malondialdehyde (MDA) was measured in these cells. **H** The level of malondialdehyde (MDA) was measured in BRD4 knocking down cells

ROS is a significant feature of ferroptosis in cells. Therefore, we continued to study whether the promotion of BRD4 inhibitors on ferroptosis of HEK293T and HeLa cells was related to ROS. We found that ROS increased significantly in HEK293T cells after treatment with JQ-1 (Fig. 3A). The ROS accumulation induced by JQ-1 was greater than that of the positive control, which was treated by Rosup. Another inhibitor, I-BET-762, showed a similar effect on ROS production (Fig. 3B). In HeLa cells, we also observed a similar effect on ROS production (Fig. 3C and 3D). To further validate the finding, we examined the ROS level in stable *BRD4* knockdown cells. Compared to control cells, the stable *BRD4* knockdown cells showed higher levels of ROS (Fig. 3E and 3F). Excess of ROS could attack biomembranes to induce lipid peroxidation. Thus, we examined the cellular level of malondialdehyde (MDA), which is a marker for lipid peroxidation and ferroptosis. Our results showed that JQ-1 or I-BET-762 treatment could significantly increase the level of cellular malondialdehyde **(**Fig. 3G**)**. Furthermore, the level of MDA was also increased in BRD4 knocking down cells compared to control cells **(**Fig. 3H**)**. These studies showed that BRD4 inhibitors induced the accumulation of ROS in both HEK293T and HeLa cells. The results suggest that BRD4 inhibitors may commonly trigger ROS and thus promote ferroptosis.

### Effect of BRD4 inhibitors on VDAC2 and VDAC3 in HEK293T and HeLa cells

**Fig. 4:**
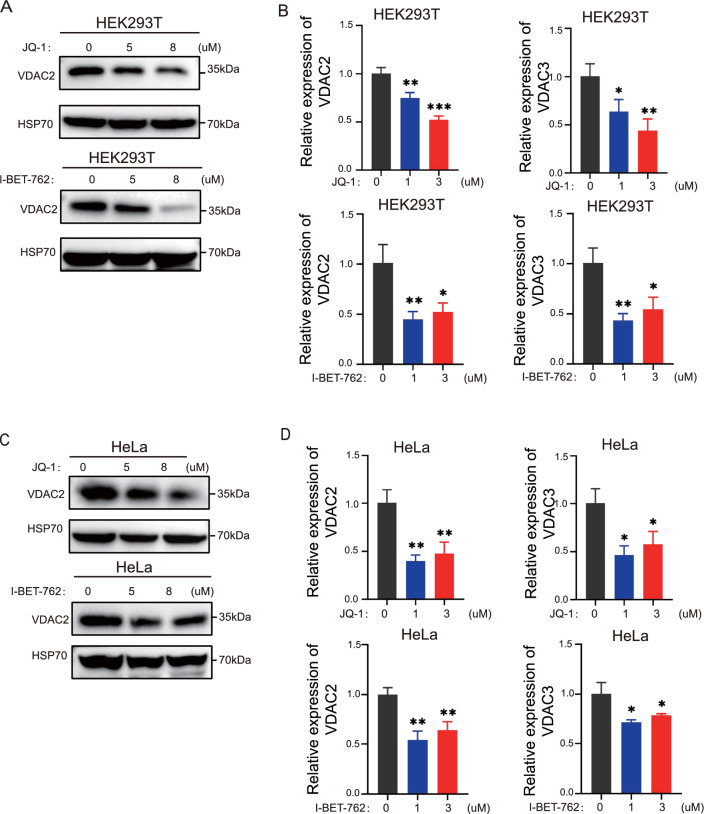
Effect of BRD4 on VDAC2 and VDAC3 in HEK293T cells and HeLa cells. HEK293T cells and HeLa cells were treated with JQ-1 and I-BET-762 for 24 h. **A** The protein level of VDAC2 in HEK293T cells was detected by Western blot. **B** qRT-PCR analysis of the mRNA expression levels of VDAC2 and VDAC3 in HEK293T cells. **C** The protein levels of VDAC2 in HeLa cells were detected by Western blot. **D** qRT-PCR analysis of the mRNA expression levels of VDAC2 and VDAC3 in HeLa cells. *p < 0 .05; **p < 0 .01; ***p < 0.001

VDAC is an ion channel on the mitochondrial membrane that promotes ROS in mitochondria to enter the cytoplasm [4, 14]. The upregulation of ROS in cytoplasm mediated by VDAC2 and VDAC3 is one of the mechanisms by which erastin promotes ferroptosis in cells [4, 15]. However, we found that inhibition of BRD4 led to the downregulation of the expression of VDAC2 in HEK293T cells (Fig. 4A). At the same time, a BRD4 inhibitor also significantly reduced the mRNA levels of *VDAC2* and *VDAC3* (Fig. 4B). Similarly, the inhibition of BRD4 resulted in a downregulation of VDAC2 in HeLa cells (Fig. 4C). BRD4 inhibitors also significantly repressed the mRNA of *VDAC2* and *VDAC3* in HeLa cells (Fig. 4D). These experiments demonstrated that although BRD4 inhibitors significantly reduced the level of VDAC2 and VDAC3, they could not explain the greatly increased ROS production upon BRD4 inhibition.

### Effect of BRD4 inhibitors on Nrf2 in HEK293T and HeLa cells

**Fig. 5:**
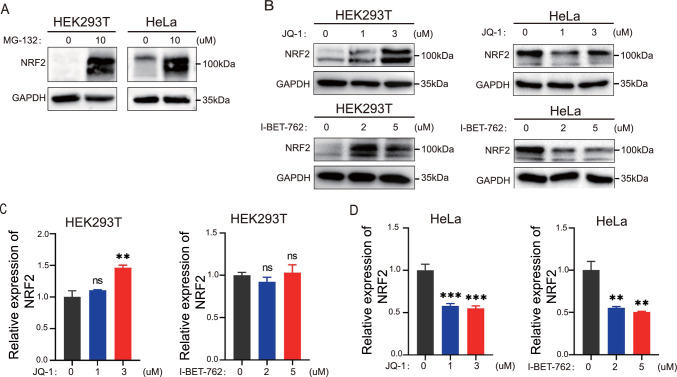
Effect of BRD4 on Nrf2 in HEK293T cells and HeLa cells. **A** Western blot was used to detect the protein level of Nrf2 in HEK293T cells and HeLa cells with MG132 treatment. HEK293T cells and HeLa cells were treated with JQ-1 and I-BET-762 for 24 h. **B**NFR2 protein levels were measured by Western blot. NRF2 mRNA levels in HEK293T cells (**C**) and HeLa cells (**D**) were determined by qRT-PCR. **p < 0 .01; ***p < 0.001

To further explore the specific mechanism by which BRD4 limited ROS production, we analyzed the expression of antioxidant Nrf2. Due to the presence of non-specific bands after incubation with the NRF2 antibody, we used the proteasome inhibitor MG132 to inhibit the ubiquitination degradation of NRF2 to determine the specific bands of NRF2 (Fig. 5A). We further examined the changes of NRF2 protein and mRNA levels in cells after treatment with BRD4 inhibitors JQ-1 and I-BET-762. The results showed that the Nrf2 protein levels in HEK293T cells increased significantly, but were significantly downregulated in HeLa cells (Fig. 5B). The changes in *NRF2* mRNA levels in HEK293T and HeLa cells were similar to the changes in protein levels after treatment with JQ-1 or I-BET-762 (Fig. 5C and 5D). This suggests that BRD4 inhibitor-induced ROS production was not affected by NRF2.

### Effect of BRD4 inhibitors on system Xc and GPX4 in HEK293T and HeLa cells

**Fig. 6:**
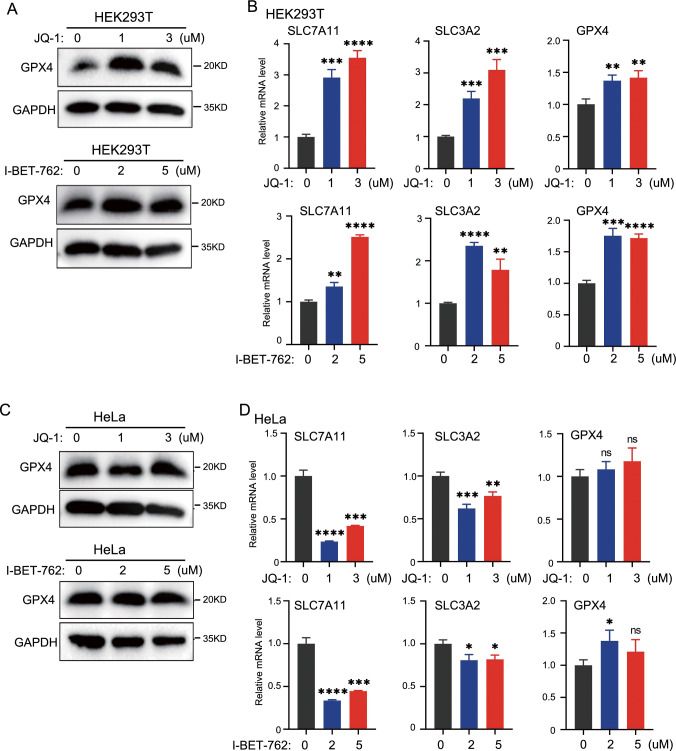
Effect of BRD4 on System Xc and GPX4 in HEK293T cells and HeLa cells. HEK293T cells and HeLa cells were treated with JQ-1 and I-BET-762 for 24 h. **A** The protein level of GPX4 in HEK293T cells was detected by Western blot. **B** qRT-PCR analysis of the mRNA expression levels of SLC7A11, SLC3A2, and GPX4 in HEK293T cells. **C** Detected the protein levels of GPX4 in HeLa cells by Western blot. **D** The mRNA expression levels of SLC7A11, SLC3A2, and GPX4 in HeLa cells were measured by qRT-PCR. *p < 0 .05; **p < 0 .01; ***p < 0.001

GPX4 and system Xc, which includes SLC7A11 and SLC3A2 subunits, are important mechanisms for inhibiting ROS production and ferroptosis [1, 2, 16]. The protein level of GPX4 was slightly increased in HEK293T cells after JQ-1 treatment (Fig. 6A). The mRNA levels of *SLC7A11*, *SLC3A2*, and *GPX4* in HEK293T cells were elevated after treatment with BRD4 inhibitors (Fig. 6B). However, the protein level of GPX4 in HeLa cells was not affected by BRD4 inhibitors (Fig. 6C). In HeLa cells, BRD4 inhibition significantly reduced the mRNA levels of *SLC7A11* and *SLC3A2*, but BRD4 inhibition had minimal effect on the *GPX4* mRNA level (Fig. 6D). This indicated that BRD4 inhibitors did not induce ROS production by decreasing the protein level of GPX4, SLC7A11, or SLC3A2 in either HEK293T or HeLa cells Additional file 2: Figure S2.

### BRD4 inhibitors decreased the level of FSP1 in HEK293T and HeLa cells

**Fig. 7:**
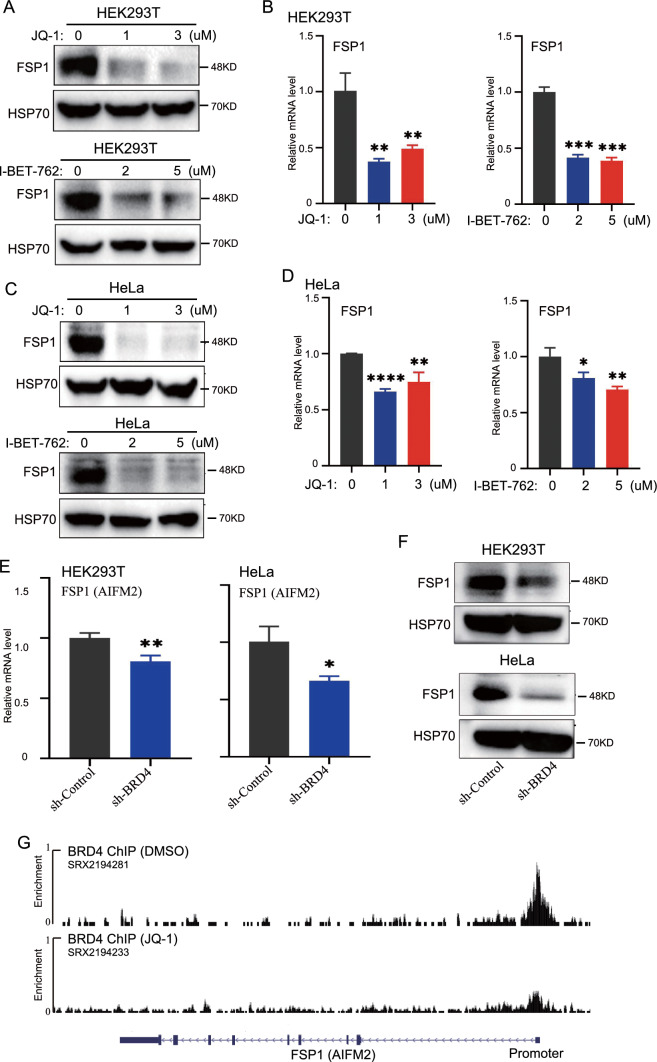
Effect of BRD4 on the expression of FSP1 in HEK293T cells and HeLa cells. HEK293T cells and HeLa cells were treated with JQ-1 and I-BET-762 for 24 h. The protein level of FSP1 in (**A**) HEK293T cells and (**C**) HeLa cells were detected by Western blot. The mRNA expression levels of FSP1 in (**B**) HEK293T cells and (**D**) HeLa cells were measured by qRT-PCR. **E** The mRNA levels of FSP1 in control and stable BRD4 knockdown cells in HEK293T and HeLa cells were measured by qRT-PCR. **F** The protein levels of FSP1 in control and stable BRD4 knockdown cells in HEK293T and HeLa cells were measured by Western blot. **G** The ChIP sequencing data of BRD4 in SUM-159 cells with (SRX2194233) or without JQ-1(SRX2194281) treatment was download from GEO dataset and the data was analyzed by using USCS genome. HeLa cells were measured by qRT-PCR.*p < 0 .05; **p < 0 .01; ***p < 0.001

FSP1 is part of another GPX4-independent mechanism for inhibiting ROS production in ferroptosis [17]. Thus, we examined whether BRD4 inhibition affected FSP1 expression in cells. Importantly, JQ-1 treatment in HEK293T cells greatly reduced the protein level of FSP1 (Fig. 7A). Consistently, I-BET-762 also significantly reduced the protein level of FSP1 (Fig. 7A). Furthermore, the mRNA level of *FSP1* was also greatly reduced upon JQ-1 and I-BET-762 treatment (Fig. 7B). Next, we determined the effect of BRD4 inhibition on FSP1 in HeLa cells. Consistent with the results from HEK293T cells, BRD4 inhibition greatly reduced the protein and mRNA level of FSP1 in HeLa cells (Fig. 7C and 7D). To further validate these results, we determined the level of FSP1 in control and stable *BRD4* knockdown cells. The mRNA level of *FSP1* was significantly reduced in stable *BRD4* knockdown cells (Fig. 7E). Consistent with the downregulation of *FSP1* mRNA, the protein level of FSP1 was also greatly reduced (Fig. 7F). To further confirm the direct regulation of BRD4 on FSP1, we analyzed the published BRD4 ChIP-sequencing data. In the *FSP1* (AIFM2) promoter, we found significant enrichment of BRD4. Furthermore, the binding of BRD4 at the *FSP1* promoter was greatly reduced (Fig. 7G). The results demonstrated a critical role of BRD4 on the expression of FSP1. To further confirm the effect of BRD4 inhibition on the exppression of FTH1, FTL, VDAC2, VDAC3, NRF2 and GPX4, we re-analyzed a large dataset from Connectivity Map, which tested ~ 5000 small-molecule compounds in 36 cell lines. Their tested compounds including I-BET-762 which is a compound used in our manuscript. We analyzed the expression of NFE2L2, FTH1, FTL, GPX4 and VDAC2 after treatment with I-BET-762 in 36 cell lines (Additional file 3: Figure S3). Consistent with our results, the expression of NFE2L2, FTH1, FTL, GPX4 and VDAC2 after treatment with I-BET-762 was inconsistent changed (Supplemental Figure S3A, S3B). In most cell lines, the expression of NFE2L2, FTH1, FTL, GPX4 and VDAC2 after treatment with I-BET-762 was up-regulated. By considering the consistent effect of BRD4 inhibition on FSP1, our findings suggest that BRD4 inhibition promotes ferroptosis very likely through FSP1.

## Materials and methods

### Cell culture and treatment

HEK293T cells (BNCC353535, BNCC) and HeLa cells (BNCC342189, BNCC) were cultured in DMEM (SH30243.01; HYClone) medium with 10% bovine fetal serum (03.C16001DC; EarlBio) and 1% penicillin–streptomycin antibiotic mixture (P1400; Solarbio) in an incubator with 5% CO^2^ under 37 °C. HepG2, RKO and PC3 were also cultured in DMEM with 10% FBS. To establish BRD4 overexpression cell lines, plasmid encoding BRD4 were transfected into HEK293T cells. After 48 h, cells were collected or indicated drugs were added. To generate stable BRD4 knockdown cells, HEK293T and HeLa cells were transfected with shRNAs targeting on BRD4. After 48 h, the transfected cells were selected by puromycine for two weeks. As for drug treatment, drugs of different concentrations were added after the cells adhere to the cell culture dishes and recover their shape. The drugs include Erastin (B1524, APExBIO), JQ-1 (A1910, APExBIO), I-BET-762 (B1498, APExBIO), and MG132 (A2585, APExBIO).

### Propidium iodide staining

The propidium iodide staining was used to assess cell death. HEK293T cells and HeLa cells were treated with DMSO, JQ-1 (1 μM), I-BET-762 (2 μM), Erastin (20 μM), and Erastin with JQ-1 or I-BET-762 respectively for 48 h. HepG2, RKO, PC3 cells and stable BRD4 knockdown cells were also treated as indicated drugs. Then the cells were treated with 5 ug/mL propidium iodide (P3566, Thermo Fisher Scientific) for 15 min. The samples were photographed to observe cell morphology and death.

### Assessment of cell viability

The CCK-8 (BMU106-CNA, Abbkine) was used to assess the cell viability of cells. HEK293T cells and HeLa cells were seeded in a 96-well plate with 100 μL serum free medium. Then, 10 μL CCK-8 was added for further cultivation for 2 h. The OD value of each orifice was measured at 450 nm. The cell viability of each group was calculated from the percentage of the DMSO group.

### RNA isolation and quantitative real-time PCR

The Trizol method was used to extract total RNA, and its concentration was detected by the nucleic acid tester. cDNA products were synthesized using HiScript III RT SuperMix for qPCR (+ gDNA wiper) kit (R323-01; Vazyme). qPCR was carried out using the AceQ qPCR SYBR Green Master Mix kit (Q111-02; Vazyme). The primers used for qPCR are listed in Supplementary Table. Next, the relative expression levels of genes were calculated by using GAPDH as an internal control.

### Western blot

Collected cells were homogenized in RIPA lysis buffer with 1 mM PMSF (R0020; Solarbio). The cells were crushed three times with an ultrasonic vibrator for 10 s each time. After the cells were treated on the ice for 30 min, centrifuged at 12,000 rpm at 4 °C for 10 min, and the supernatants were collected. The proteins were electrophoresed with 10% SDS-PAGE gel, then transferred the protein on the gel was to the polyvinyl-idene fluoride (PVDF) membrane. The PVDF membrane was blocked in TBST containing 5% milk for 1 h. The primary antibody (1:1000) was incubated overnight. The main primary antibodies were VDAC2 (A18683; ABclonal), FSP1 (342551; ZEN-BIO), NRF2 (A0674; ABclonal), GPX4 (sc-166570; Santa Cruz), FTH1 (sc-376594; Santa Cruz), FTL (sc-74513; Santa Cruz)), GAPDH (sc-365062; Santa Cruz), HSP70 (sc-24; Santa Cruz). The membrane was washed three times with TBST buffer and incubated with horseradish peroxidase-conjugated secondary antibody (1:2000) for 2 h at room temperature. After washing with TBST, antibody-bound proteins were detected with ECL electrochemiluminescence reagent (BL520B, Biosharp).

### Assessment of ROS and MDA level in cells

Reactive oxygen species assay kit (CA1410; Solarbio) was used to assess the ROS level in cells. HEK293T cells and HeLa cells as well as stable BRD4 knockdown cells were treated with DMSO or BRD4 inhibitors for 24 h respectively. Rosup, a reagent that can stimulate cells to produce ROS, was diluted to 50 ug/mL by medium, and then added into the cells of Rosup group for 40 min. Then all cells of each group were washed twice with medium and the remaining liquid was sucked away. 100 μL ROS probe DCFH-D with a concentration of 5 μmol/L was added to cells of each group and incubated in a 37 ℃ cell incubator for 20 min. The cells were washed 3 times with serum-free culture solution. Fluorescence intensity was measured at excitation light 488 nm and emission light 525 nm. MDA  levels were  measured following the instructions of  the MDA test kit (BC0025, Solarbio).

### Dataset analysis from chemical treated cell lines

We downloaded gene expression files for inhibitor-treated cell lines (level3_beta_trt_cp_n1805898 × 12,328.gctx) and DMSO-treated cell lines (level3_beta_ctl_n188708 × 12,328.gctx) from the cmap database (https://clue.io/data/CMap2020#LINCS2020). Using the cmapR package in RStudio, we extracted the expression files for five genes (NFE2L2, FTH1, FTL, GPX4, VDAC2) for cell lines treated with I-BET-762 and their corresponding DMSO-treated groups. We filtered the treatment groups to include only cell lines treated with DMSO, 1.11 μM, 3.33 μM, and 10 μM, as well as DMSO, 0.66 μM, 2.5 μM, and 10 μM. Subsequently, we created line plots of the expression of the target genes for cell lines treated with I-BET-762 and their corresponding DMSO-treated groups using the ggplot2 package in R.

### Statistical analysis

All data were expressed as mean ± standard error. Statistical analyses were per-formed using the GraphPad Prism 8.0. One-way analysis of variance was used to com-pare multiple groups. p < 0.05 was considered statistically significant.

## Discussion

Ferroptosis is a unique means of regulating cell death, driven by iron-dependent phospholipid peroxidation and regulated by many factors, including iron homeostasis, oxidative stress, and lipid metabolism [2]. Ferroptosis promotes the development of a variety of diseases, and is a promising strategy for cancer treatment [5]. BRD4 is an important regulator in tumor development. JQ-1, an inhibitor of BRD4, was found to promote ferroptosis of cancer cells and inhibit tumor development [6]. However, a recent study revealed that JQ-1 blocked erastin-induced ferroptosis [11]. Thus, the effect of BRD4 inhibition on ferroptosis needs to be further explored in more cancer cell lines. We found that the BRD4 inhibitor JQ-1 and I-BET-762 promoted erastin-induced ferroptosis and decreased cell viability in a panel of five cell lines, HEK293T, HeLa, HepG2, RKO, and PC3. After using the BRD4 inhibitors, JQ-1 and I-BET-762, the expression of FTH1, ROS, Nrf2, and GPX4 increased in HEK293T cells, while the expression of VDAC2, VDAC3 and FSP1 decreased. JQ-1 and I-BET-762 resulted in a decrease in the expression of FTH1, VDAC2, VDAC3, Nrf2, GPX4, and FSP1 in HeLa cells, accompanied by an increase in the expression of ROS. In stable *BRD4* knockdown cell lines, we also found an increased ROS production and FSP1 downregulation. These results showed that the promotion of ferroptosis by BRD4 inhibitors in HEK293T cells was related to the downregulation of FSP1 to increase ROS. Meanwhile, the promotion of ferroptosis by BRD4 inhibitors in HeLa cells was associated with decreased expressions of FTH1, NRF2, and FSP1, as well as increased expression of ROS. Our results suggest that BRD4 inhibitors have different effects on ferroptosis-associated genes in different cell lines. However, ROS accumulation and FSP1 downregulation upon treatment with BRD4 inhibitors were common mechanisms. Thus, BRD4 inhibitors might be more valuable in combination with ferroptosis inducers in FSP1-dependent cancer cells. This hypothesis should be further explored.

Erastin is a small molecule compound that is widely used as an inducer of ferroptosis in cells, such as engineered cancer [4], melanoma [15], ectopic endometrial stromal [18], and HEK293T cells [19]. Erastin significantly reduced ferroptosis in HeLa cells [20]. BRD4 is a member of the BET family and plays a key role in regulating cell growth and cancer development by binding to acetylated lysine residues of histones and other proteins [21]. BRD4 reduces ferroptosis in neurons and oligodendrocytes [22]. BRD4 protein contains two subtypes, BD1 and BD2, with high sequence similarity [5]. Therefore, it is challenging to obtain a selective inhibition for each bromodomain in the BET family. BET inhibitors, such as JQ-1 and I-BET-151, are widely used to study the function of BET proteins by simultaneously inhibiting multiple bromodomains [5]. JQ-1 treatment or *BRD4* knockdown led to ferroptosis in MDA-MB-231, A549, and Hs578T cells, accompanied by an increase of ROS and iron levels, and the collaborative treatment of JQ-1 and erastin aggravated these changes [6]. We found that erastin combined with JQ-1 or I-BET-762 significantly enhanced ferroptosis and further reduced cell viability in five cancer cell lines, including multiple cancer types. Therefore, it has great potential to promote the ferroptosis of cancer cells and inhibit tumor development through the cooperation of BRD4 inhibitors and erastin.

GPX4 and FSP1 are two classical signal pathways that inhibit ferroptosis through ROS generation. System Xc, a dimer composed of SLC7A11 and SLC3A2, generates a powerful reducing agent, glutathione (GSH), by transferring extracellular cystine into cells [10]. GSH, as a cofactor of GPX4, promotes the reduction of PLOOHs to PLOHs in the cell, reducing ROS accumulation [23]. The levels of GPX4, SLC7A11, and SLC3A2 were reduced in cancer cells treated with either *BRD4* knockout or JQ-1 [6]. However, our study showed that neither BRD4 inhibitor studied here decreased GPX4 expression in HEK293T or HeLa cells. This suggests that JQ-1 and I-BET-762 do not promote the generation of ROS in HEK293T and HeLa cells through GPX4 or through SLC7A11 and SLC3A2. FSP1 is independent of GPX4 and inhibits ferroptosis by inhibiting lipid peroxidation [1, 2]. FSP1 acts by catalyzing the reduction of ubiquinone to panthenol, which stops the lipid autoxidation reaction [4]. FSP1 also catalyzes the reduction of the oxidized state alpha-tocopherol to its non-free radical form, producing the most potent and natural chain-blocking antioxidant in lipids [4]. We found that the expression of FSP1 in HEK293T cells and HeLa cells was significantly reduced after treatment with JQ-1 or I-BET-762. The results demonstrate a critical role of BRD4 on the expression of FSP1. The regulation of BRD4 on FSP1 was validated by stable BRD4 knockdown cells. Furthremore, we also found BRD4 binds the promoter of FSP1. By considering the common effect of BRD4 inhibition on FSP1, it suggests that BRD4 inhibitors are widespread in promoting ferroptosis through FSP1. However, the specific mechanism of BRD4 promoting the expression of FSP1 deserves further study.

## Conclusions

In conclusion, we found that BRD4 inhibitors significantly increased erastin-induced ferroptosis in multiple cell types. BRD4 inhibitor-promoted ferroptosis in HEK293T cells was associated with downregulation of FSP1, increasing ROS production. BRD4 inhibitor-promoted ferroptosis in HeLa cells was associated with increased Fe^2+^ levels caused by decreasing the level of FTH1, and increased ROS levels caused by the decreasing levels of NRF2 and FSP1. ROS accumulation and FSP1 downregulation were found to be the common mechanisms underlying increased ferroptosis with BRD4 inhibitors. BRD4 inhibitors have great potential to promote ferroptosis in cells, especially in cancer cells treated with erastin. Therefore, BRD4 inhibitors are expected to become a target for inhibiting tumor growth.

### Supplementary Information

**Additional file 1.Figure S1** The BRD4 inhibitor I-BET-762 promoted ferroptosis induced by erastin in a panel of five cell lines, HEK293T, HeLa, HepG2, RKO, and PC3.

**Additional file 2.Figure S2** Stable cell lines were established by knocking down BRD4 in HEK293T and HeLa cells.

**Additional file 3.Figure S3** The expression of NFE2L2, FTH1, FTL, GPX4 and VDAC2 after treatment with I-BET-762 in 36 cell lines

**Additional file 4.Table S1** The primer sequences used for real-time PCR.

## Data Availability

The data presented in this study are available on request from the corresponding author upon a reasonable request.
